# A New Method of Assessing Sheep Red Blood Cell Types from Their Morphology

**DOI:** 10.3390/ani9121130

**Published:** 2019-12-12

**Authors:** Ivona Žura Žaja, Silvijo Vince, Nina Poljičak Milas, Ingo Ralph Albin Lobpreis, Branimira Špoljarić, Ana Shek Vugrovečki, Suzana Milinković-Tur, Miljenko Šimpraga, Luka Pajurin, Tomislav Mikuš, Ksenija Vlahović, Maja Popović, Daniel Špoljarić

**Affiliations:** 1 Department of Physiology and Radiobiology, Faculty of Veterinary Medicine, University of Zagreb, Heinzelova 55, 10000 Zagreb, Croatiaana.shek@vef.hr (A.S.V.); tur@vef.hr (S.M.-T.);; 2 Clinic for Reproduction and Obstetrics, Faculty of Veterinary Medicine, University of Zagreb, Heinzelova 55, 10000 Zagreb, Croatia; svince@vef.hr; 3 Department of Pathophysiology, Faculty of Veterinary Medicine, University of Zagreb, Heinzelova 55, 10000 Zagreb, Croatia; nmilas@vef.hr; 4 Faculty of Veterinary Medicine, University of Zagreb, Heinzelova 55, 10000 Zagreb, Croatia; ilobpreis@vef.hr; 5 Department of Veterinary Biology, Faculty of Veterinary Medicine, University of Zagreb, Heinzelova 55, 10000 Zagreb, Croatia; lpajurin@vef.hr (L.P.); vlahovic@vef.hr (K.V.); maja.popovic@vef.hr (M.P.); daniel.spoljaric@vef.hr (D.Š.); 6 Department of Hygiene, Technology and Food Safety, Faculty of Veterinary Medicine, University of Zagreb, Heinzelova 55, 10000 Zagreb, Croatia; tmikus@vef.hr

**Keywords:** RBC subpopulations, haematological parameters, Lika pramenka sheep, morphometric analysis

## Abstract

**Simple Summary:**

Haematological tests are an important diagnostic tool for animal diseases. However, little is known beyond the standardly used haematological methods in sheep, especially those in which the detection of sheep red blood cells (RBCs) shape changes is crucial. Our goal is to obtain sheep RBC morphometric parameters as well as RBC subpopulations based on their morphometric parameters. Morphometric parameters of RBC size and shape were determined from stained blood smears using SFORM, a computer-assisted program for automated cell morphometric measurements. Based on their morphometric parameters, three RBC subpopulations were obtained using principal component and cluster analysis: the smallest and most elongated RBCs, the biggest and most rounded ones, and RBCs of average size and shape. When the values of RBC haematological parameters were higher or above the physiological range, a significantly higher proportion of both average size and shape RBCs, as well as the biggest and most rounded RBCs, was obtained. Since they were obtained from healthy animals, these results indicated the importance of determining morphometric parameters of RBCs and the proportion of each RBC subpopulation, which could serve as a basis for future possibilities in the diagnostic interpretation of haematological disorders in sheep, especially those for which the detection of shape changes in ovine RBCs is crucial.

**Abstract:**

Data concerning the morphometric parameters of sheep red blood cells (RBCs) obtained using computer-assisted image analysis have not yet been investigated, and there are no data on any analyses of ovine RBC subpopulations based on their morphometric parameters. The aims of this study are to determine the values of RBC haematological and morphometric size and shape parameters, to form groups according to the obtained values of haematological parameters; to determine the differences in RBC morphometric parameters between the formed groups, and to determine RBC subpopulations and their respective proportions in the formed groups. Thirty-six blood samples were collected from the jugular vein of clinically healthy Lika pramenka sheep, aged between 2 and 5 years. Haematological parameters including haemoglobin (HGB), haematocrit (HTC), mean corpuscular volume (MCV), mean corpuscular haemoglobin (MCH), mean corpuscular haemoglobin concentration (MCHC), and RBC distribution width were analysed using a haematology analyser. Haematological parameters were categorized into two groups: those with lower values or values below the physiological range (Groups 1) and groups with higher values or values above the physiological range (Groups 2). Morphometric parameters of RBCs were determined from stained blood smears using SFORM, a computer-assisted program. Significantly higher values of RBC area, outline, convex, minimal and maximal radius, as well as length and breadth were established in Groups 2 compared to Groups 1 of HGB, HCT, MCV, MCH, and MCHC, respectively. Based on the morphometric parameters of RBCs, three RBC subpopulations were obtained using principal component and cluster analysis: ES 1—the smallest and most elongated RBCs, ES 2—the biggest and most rounded RBCs, and ES 3—average size and shape RBCs. Significantly higher proportions of ES 2 and ES 3 subpopulations, as well as a significantly lower proportion of ES 1 subpopulation, were established in Groups 2 compared to Groups 1 of HGB, HTC, MCV, and MCH, respectively. It can be concluded that ovine RBC subpopulations, based on their morphometric parameters, can be obtained by using computer-assisted image analysis of RBC morphometry and multivariate statistical methods, including principal component and cluster analysis. RBC morphometry, including classification into subpopulations, could serve as a basis for future possibilities in the diagnostic interpretation of anaemic syndromes in veterinary medicine, especially in normocytic, macrocytic, and microcytic anaemias in sheep.

## 1. Introduction

The Lika pramenka breed is the most numerous autochthonous population of sheep in Croatia [1]. In recent years, there has been a trend of some intensification of management regarding health control of sheep flocks. Together with anamnestic data, clinical examination, and other diagnostic tools, haematological profile presents a valuable tool for achieving final diagnoses [2]. It is already known that, due to their small size, ovine red blood cells (RBCs) are often erroneously interpreted when analysed on haematology instruments calibrated for other species [2].

Until recently, morphometric studies of RBCs were essentially based on linear measures of RBC size. Use of an ocular micrometer and a lens micrometer (micrometric slide) is the only valid and recognised method of measuring the size of RBCs [3]. Morphometric studies of RBCs performed by sophisticated and advanced software are more appropriate and more precise than conventional measurements with an ocular micrometer. This software provides a numerical objectification of the most subtle modifications unable to be estimated visually, and as such, has clinical and research applications that are becoming more numerous, especially in cytology and histopathology [3,4,5,6]. Morphometry is the simplest form of image cytometry and refers to the evaluation of cells or tissue by measuring various cellular features in a two-dimensional view [5]. Furthermore, changes in RBC morphometric indicators have been detected in certain human [4,7,8] and dog ailments [9]. Morphometric studies of RBCs showed several morphological changes; the most important one is due to age and breed in sheep and goats [10], and also in fish [11]. Buslovskaya et al. [12] defined a set of changes of several morphometric and functional parameters of RBCs and white blood cells, characteristic of different adaptive reactions of the chicken organism.

RBC senescence has so far been investigated through the isolation of RBC populations of different mean cell ages. Most of the research has been conducted on RBCs separated on the basis of differences in cell density or volume/size [13,14]. Of the various techniques used, only a handful have found extensive application in basic science studies: plain centrifugation, angle-head centrifugation, and the use of several discontinuous gradients, which result in variably efficient separation. The use of a Percoll gradient has proven to be an easy and efficient way of separating RBCs [13,14,15]. Nevertheless, it has been suggested that density is not a good criterion to determine RBC age, and it has been proposed that separation exploiting differences in RBC volumes through counterflow centrifugation might yield better results. Studies have been conducted over the years addressing the peculiar characteristics of RBC subpopulations, from younger to older fractions. RBC aging has been reported to correlate with decreased cell volume, size and mean corpuscular volume [14,16], increased mean corpuscular haemoglobin concentration [14] and cell deformability [15].

Data concerning the morphometric size and shape parameters of sheep RBCs obtained using computer-assisted image analysis have not been investigated. In addition, to the best of our knowledge, there are no data in recent literature on any analyses of ovine RBC subpopulations with respect to morphometric size and shape parameters, both approaches using computer-assisted image analysis of RBC morphometry and multivariate statistical methods, including discriminant and cluster analyses. The hypothesis of this research is based on the assumption that there might be a connection between RBC morphometric and haematological parameters, as well as differences in the distribution of RBC subpopulations in categorised groups according to the obtained haematological parameters’ values in Lika pramenka sheep. Therefore, the aims of this study are to determine the values of RBC haematological and morphometric size and shape parameters, to form RBC groups according to the obtained values of haematological parameters, to determine the differences in morphometric parameters between the formed RBC groups and to determine RBC subpopulations and their respective proportions in the formed groups.

## 2. Materials and Methods

### 2.1. Ethics and Welfare Approval Statement

All procedures used in this research were in compliance with the European guidelines for the care and use of animals in research (Directive 2010/63/EC) and approved by the Committee for Ethics in Veterinary Science, Faculty of Veterinary Medicine, University of Zagreb, Croatia (records No.: 640-01/16-17/54; file No.: 251-61-01/139-16-2) and Veterinary and Food Safety Directorate, Ministry of Agriculture, Republic of Croatia (records No.: UP/I-322-01/17-01/31; file No.: 525-10/0529-17-2).

### 2.2. Animals. The Research is Part of the Project

“Innovative functional lamb meat products” financed by the Croatian Science Foundation (IP-2016-06-3685). It was conducted on a sheep farm of Lika pramenka breed, in the ownership of GEA-COM Ltd., situated in Crkvina, Krnjak area, Croatia. The farm is situated at an altitude of 215 m above sea level and is composed of 200 sheep and 20 rams of varying age.

The animals are kept extensively, grazing in the field in the warmer periods of the year, and in a barn during winter, when they are fed with hay from the surrounding pastures. Independently of the season, the sheep are also fed with pelleted complete feed (KUŠIĆ PROMET Ltd., Donje, Psarjevo, Croatia product code 914106).

### 2.3. Study Design and Collection of Blood from the Sheep

The research comprised 36 randomly chosen sheep, 2 to 5 years old, with an average body weight of 37.58 ± 2.66 kg. The sheep were clinically examined during May, outside of the reproductive season, and their blood was drawn for further haematological analyses. Blood was collected by venepuncture of the jugular vein, using vacuum tubes with EDTA. Before the venepuncture, hair surrounding the puncture site was clipped off and skin was disinfected according to the usual veterinary aseptic procedure. After the venepuncture and collection of 5 mL of blood in vacuum tubes, 500 µL of blood was separated in micro test tubes (BD Microtainer^®^ Tube, K2EDTA). The samples were then stored at +4 °C and processed in the laboratory on the same day, approximately 5 h after the collection.

### 2.4. Haematological Analyses

Before the analyses, blood samples were mixed in automatic mixers for 30 min at room temperature (20 °C). Haematological analyses were performed on a fully automated counter made for in vitro use, the Abacus Junior Vet haematology analyser (Diatron, Táblás u., Hungary). All analyses were repeated two times, with a maximal error of <4%. Automated sampling was performed with authorised reagents (Daitro Lyse_DIFF, Diatro Cleanerand Diatro-Rinse) in four phases. Among standard haematological parameters, those regarding RBCs were chosen for further analyses: haemoglobin (HGB), haematocrit (HTC), mean corpuscular volume (MCV), mean corpuscular haemoglobin (MCH), mean corpuscular haemoglobin concentration (MCHC), and red blood cell distribution width (RDW). Furthermore, a smear was prepared from each sample and stained using Pappenheim’s panoptic staining method with the May–Grünwald and Giemsa stains [17,18].

### 2.5. Morphometric Analysis of RBCs

**Figure 1:**
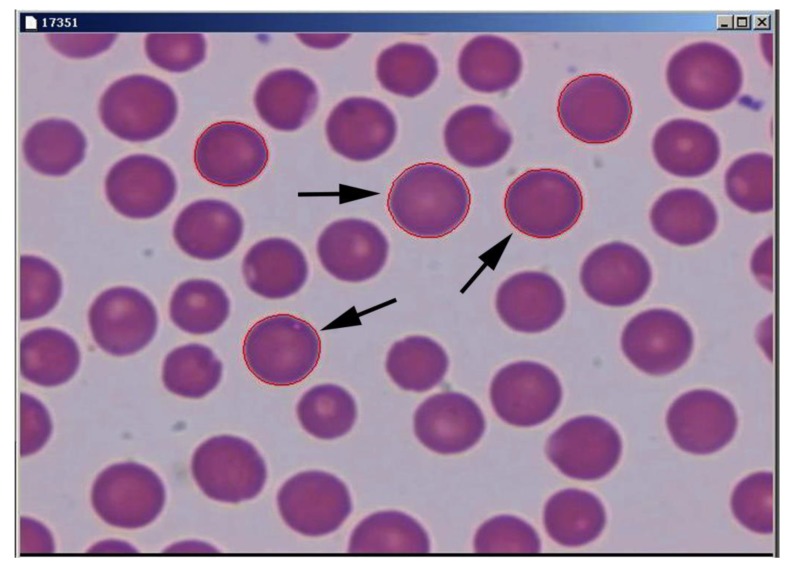
Sheep RBCs stained by Pappenheim’s method and automatically marked by the computer-assisted program SFORM (arrow: automatically marked RBC).

RBC morphometry was conducted using a computer-assisted program package for image analysis, SFORM (VAMSTEC, Zagreb, Croatia). The system consists of a high-resolution colour camera (Donpisha 3CCD colour vision camera module, Japan) that digitalises and transfers images from an Olympus BX 41 (Olympus, Tokyo, Japan) light microscope, following observation under the magnification of 1000×, onto a personal computer. Morphometric analysis was conducted on the blood smears stained according to Pappenheim’s method. Staining makes RBCs more visible and gives them contrast, which enables automated measurements with the SFORM software. A total of 36 stained blood smears were analysed, with approximately 110 RBCs per smear. Only RBCs that did not overlap with other cells or debris were measured and analysed (n = 4017). The borders of the RBCs were marked automatically using the marking option in the SFORM program, with manual corrections using a computer mouse (Figure 1). The following morphometric variables in RBC size were determined: area (μm^2^), outline (μm), convex, minimal and maximal radius (µm), length and breadth (μm). Some of the RBC morphometric size measures were used to calculate RBC shape parameters according to the following formulas: ellipticity = length/breadth; elongation = [(length−breadth)/(length + breadth)]; solidity = area/convex; roundness = (4 × area)/[π × (major axis)^2^]; form factor = [4π × area/outline^2^]; contour index = outline/√area.

### 2.6. Statistical Analyses

The results were statistically processed using the SAS 9.4 software package (Statistical Analysis Software 2002–2012 by the SAS Institute Inc., Cary, NC, USA). Descriptive statistics was made using PROC MEANS and PROC FREQ modules. The normal distribution of data was assessed with the PROC TRANSREG module. In the case of non-normality and heteroscedasticity of variances, the transformation of variables was performed by log or exponential transformation prior to analysis using a Box–Cox transformation.

Dependent variables were analysed by multivariate variance analysis (MANOVA) based on the Wilks’ lambda criterion using the GLM procedure. A multiple comparison test of the least-square means with Tukey’s correction was performed to compare each group. Groups were formed by categorizing continuous variables of the analysed values of haematological parameters (HGB, HCT, MCV, MCH, MCHC and RDW). Those parameters were categorized into two groups: those with lower values or values below the physiological range (Groups 1) and those with higher or values above the physiological range (Groups 2), bearing in mind that the groups have approximately the same number of samples each. Age as a variable was removed since it had no effect on the tested haematological or morphometric RBC parameters.

To analyse the RBC subpopulations, principal component and cluster analyses were carried out in several steps. The principal component analysis was performed using the PROC FACTOR module in order to obtain eigenvalues of morphometric variables using Kaiser’s criterion (λ ≥ 1) and to establish the number of the main components for the next analysis. The number of clusters in K-means cluster analysis was determined using the HPCLUS procedure, which selects the best k value (number of clusters or subpopulations) using the aligned box criterion value. To obtain well-separated clusters with non-random initialisation, the FASTCLUS procedure was used. To specify the mean absolute deviation criterion and to prevent outliers from distorting the results, the FASTCLUS procedure was conducted using the LEAST and STRICT options. The last cluster analysis was done with the same procedure (PROC FASTCLUS) to assign outliers and tails to clusters using zero iterations. Although cluster analysis had determined that the best number of clusters was four, one cluster was incorporated into the nearest cluster due to the low number of RBCs that formed it. To assess the differences in the distribution of RBC subpopulations between the groups, the FREQ procedure was carried out using the Hi-squared test. The level of statistical significance was set at *p* < 0.05.

## 3. Results

### 3.1. Morphometric Size and Shape Parameters of Ovine RBCs

Descriptive statistical analyses of the data on the morphometric size and shape parameters of RBCs in Lika pramenka sheep are shown in Table 1.

Morphometric parameters of ovine RBCs in the categorized groups according to the values of the haematological parameters.

The mean and standard error values of the RBC morphometric size and shape parameters of groups formed by the categorisation of the haematological parameter values in Lika pramenka sheep are presented in Table 2. Significantly higher values of RBC area, outline, convex, minimal radius, maximal radius, length, and breadth, were established in Groups 2 compared to Groups 1 of HGB, HCT, MCV, MCH and MCHC, respectively. At the same time, the value of the RBC contour index was significantly higher in Groups 1 vs. Groups 2 of HGB, MCV, MCH and MCHC, respectively. In addition, significantly higher values of RBC solidity, roundness and form factor were recorded in Groups 2 than in Groups 1 of HGB, MCV and MCH, respectively. Only the values of RBC solidity were significantly higher in the HCT 1 group than in the HCT 2 group. There were no significant differences in the values of RBC ellipticity and elongation between the two groups of observed parameters. It was found that the average values of RBC area, outline, convex, minimal radius, maximal radius, length, breadth, solidity, and roundness were significantly higher in RDW Group 2 in comparison with RDW Group 1.

### 3.2. Principal Component and Cluster Analysis Based on Morphometric Size and Shape Parameters of Ovine RBCs

Using principal component analysis prior to the clustering of RBC morphometric parameters, three characteristic components (factors) were obtained with eigenvalues greater than 1 (λ ≥ 1), which account for the 93.8% cumulative variance of the morphometric variables (Table 3). The first factor was represented by RBC size (outline, convex, area, length and breadth), and the most important value for that factor was the RBC outline. The second and third factors were focused on the RBC shape (roundness, form factor, contour index, elongation, ellipticity and solidity), and the most important RBC value for the second factor was RBC roundness, whereas the most important value for the third factor was RBC elongation. Using only one of the most important values from each factor (RBC outline, roundness or elongation), the aligned box criterion indicates that the best number of clusters is four.

**Figure 2:**
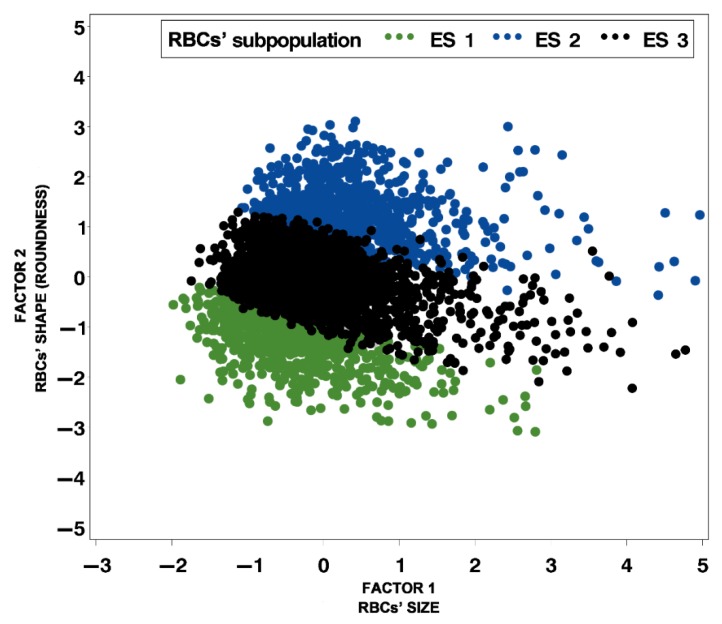
Distribution of red blood cell (RBC) subpopulations according to RBC size and shape (outline and roundness) in Lika pramenka sheep (ES 1—the smallest and most elongated RBCs; ES 2—the biggest and most rounded RBCs; ES 3—average size and shape RBCs; factor 1—outline and factor 2—roundness.

**Figure 3:**
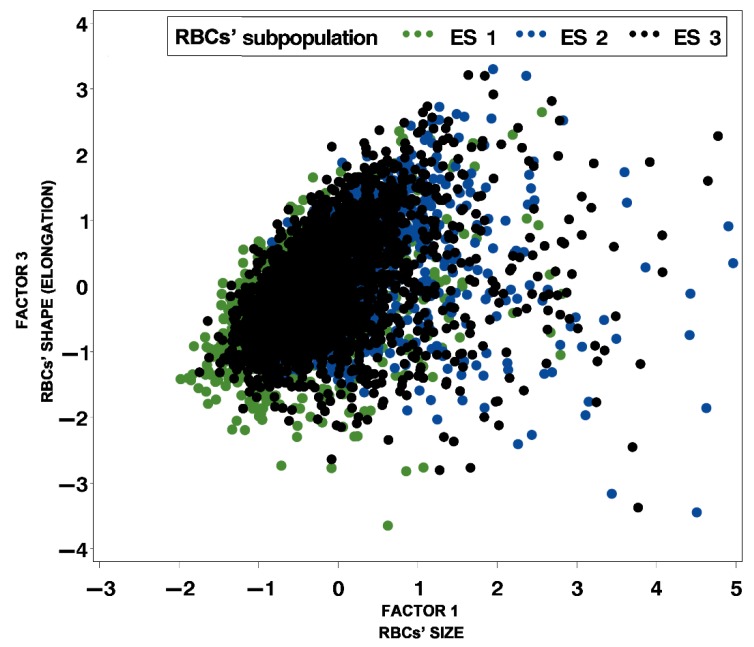
Distribution of red blood cell (RBC) subpopulations according to RBC size and shape (outline and elongation) in Lika pramenka sheep (ES 1—the smallest and most elongated RBCs; ES 2—the biggest and most rounded RBCs; ES 3—average size and shape RBCs; factor 1—outline and factor 3—elongation).

By using cluster analysis, three precisely defined RBC clusters or subpopulations were obtained based on RBC morphometric size and shape parameters (Table 4, Figure 2 and Figure 3). The first RBC subpopulation (ES 1) was comprised of the smallest and most elongated RBCs (19.5%), the second subpopulation (ES 2) comprised the biggest and most rounded ones (22.9%), and the third subpopulation (ES 3) comprised average size and shape RBCs, but with the highest proportion (57.6%). Cumulative variance of factors 1 and 2 is more than 70%, and the three subpopulations, based on the size and roundness of RBCs, are clearly visible in Figure 2. However, the explained variance of factor 3 is only 18.5%, and the differences among the subpopulations based on elongation are difficult to notice, as can be seen in Figure 3.

### 3.3. Distribution of Subpopulations of Ovine RBCs in Groups Categorized According to the Values of Haematological Parameters

By testing the distribution of RBC subpopulations according to the morphometric variables of RBC size and shape, a significant difference was observed between the groups formed by categorization of haematological parameters values (HGB, HCT, MCV, MCH, MCHC, and RDW). Significantly higher proportions of ES 2 and ES 3 subpopulations, as well as a significantly lover proportion of ES 1 subpopulation, were established in HGB 2, HCT 2, MCV 2 and MCH 2 groups compared to HGB 1, HTC 1, MCV 1 and MCH 1 groups, respectively (Table 5).

It was found that the proportions of ES 1 and ES 3 subpopulations were significantly lower while ES 2 proportion was significantly higher in the MCHC 2 group than in the MCHC 1 group. In addition, the RDW 2 group had a significantly lower proportion of ES 2 and ES 3 subpopulations and a significantly higher proportion of ES 1 subpopulation in comparison to the RDW 1 group.

## 4. Discussion

Detection of changes in RBC morphology is clinically important in haematology and medicine [20], especially as an aid in establishing a diagnosis regarding the cause of anaemia. Anaemia represents one of the more common small ruminant emergencies, which can be difficult to diagnose before it is too severe [21]. Furthermore, it is accompanied by changes in otherwise physiological anisocytosis and poikilocytosis in sheep. In this paper, these physiologic differences have been classified into three distinct RBCs’ subpopulations, based on morphometric measures. To the best of our knowledge, there is no data about RBCs’ classification into subpopulations in sheep. It is possible that the proportion and type of RBCs’ subpopulations could change, following changes in the animals’ health status. Using a combination of features of RBCs’ morphologic measures and cluster method for classification into subpopulations could enable better insight into RBCs’ abnormality analyses and shape-related diseases. However, this warrants further research.

Detection of RBC morphology is routinely performed by subjective microscopic evaluation, which is difficult and highly dependent on the operator’s expertise. Therefore, it is necessary to implement an automated methodology to analyse red blood cell shape changes to support and improve the operator’s capability and to expedite measurements [10,22]. Interpretation of RBC morphology should be carried out in conjunction with other quantitative data from the complete blood count [23]. Furthermore, haematological and biochemical tests are an important tool for the evaluation of the nutritional and health status of farm animals [2,23].

Data concerning RBC morphometric size and shape parameters in the Lika pramenka sheep breed, in general, have not been investigated, and only a few RBC morphometric size parameters (diameter, circumference, and area) in sheep have been determined using an ocular micrometer and a lens micrometer. Therefore, according to the above-mentioned results of this study, most of the investigated morphometric parameters values cannot be compared. The morphometric study of RBCs seems to show that the diameter, circumference and area of RBCs have a significant influence on the determination of the species of domestic animals [10]. So, ovine RBCs are somewhat smaller than most mammalian RBCs [2], they have a diameter of 4.5 μm, a width of 3.2–5 μm and a lifespan of 70–150 days, they do not aggregate or deform as readily as RBCs of other non-ruminant species [3,10]. In this study, the mean and standard error values for RBC length and breadth were 5.51 ± 0.47 and 4.9 ± 0.42 μm, respectively. A minor difference in the values of the mentioned morphometric parameters in this study compared to Adili et al. [3,10] is likely to be related to the different ovine breed and breeding methods. Breed, age, and sex are known to affect the values of morphometric parameters [11,24]. In our study, the values of RBC length/diameter are almost equal to those of Thamer et al. [25] (5.277 ± 0.67 μm), although in a different sheep breed. The RBC area value obtained in this study (21.19 ± 3.45 μm^2^) does not match the values reported by Adili et al. [2], whose values of RBC circumference (16.89 ± 0.72 μm) and area (16.49 ± 0.97 μm^2^), obtained from both ewes and rams, were almost equal, which is surprising.

In this research, the differences in the mean values of RBC morphometric size and shape parameters were determined between groups formed by categorization of haematological parameters values (HGB, HCT, MCV, MCH, MCHC, and RDW) in Lika pramenka sheep. In addition, significantly higher values of RBC area, outline, convex, minimal radius, maximal radius, length, and breadth, were established in the groups with higher vs. the groups with lower values of HGB, HCT, MCV, MCH, MCHC, respectively. The obtained results of RBC morphometric size parameters determined in this study are consistent with those of Ravi Sarma [26], who stated that higher values of red cell MCV, HGB, HCT, MCH, and MCHC are correlated with larger RBC size. The typical RBC shape for domestic animal species is a disc or a biconcave disc (discoid), resulting in a high surface area to volume ratio, making red blood cells deformable [3]. According to the previously mentioned morphometric shape parameters of RBC solidity, roundness, and form factor with values close to 1, the significantly higher values of RBCs in this study indicate a circular shape, which is not, according to the above, a preferred feature of RBC morphology. This fact could be explained by the physiological presence of relatively small RBCs in small ruminants compared to other species [2]. However, due to significant differences in RBC morphology in different species and the scarcity of data in this field, further studies are required to properly interpret the values of RBC morphometry parameters obtained in this study.

It has long been known that RBCs comprise various subpopulations, which can, among others, be separated through Percoll density gradients [15]. However, to the best of our knowledge, there are no data in recent literature on the analysis of RBC subpopulations in sheep with respect to morphometric size and shape parameters. In this study, by using cluster analysis, three RBC subpopulations were obtained based on RBC morphometric size and shape parameters, with the highest proportion of average size and shape RBC subpopulation (57.6%). In addition, a difference in the distribution of RBC subpopulations between the groups formed by categorisation of haematological parameters values (HGB, HCT, MCV, MCH, MCHC, and RDW) was established. The groups with higher values of HGB, HTC, MCV, and MCH comprised a significantly higher proportion of RBCs of average size and shape, as well as the subpopulation of the biggest and most rounded RBCs. According to several authors, human RBC ageing correlates with decreased cell volume, size, and mean corpuscular volume, increased mean corpuscular haemoglobin concentration and cell deformability [14,15]. Therefore, it could be concluded that the groups with higher values of HGB, HTC, MCV, and MCH obtained in this study, with a significantly higher proportion of the subpopulation of average size and shape RBCs as well as the subpopulation of the biggest and most rounded RBCs, also contained a higher proportion of younger RBCs. In addition, the group with a higher RDW value had a significantly lower proportion of both the biggest and most rounded as well as average size and shape RBC subpopulations, and a significantly higher proportion of the smallest and most elongated RBC subpopulation, in comparison to the group with a lower RDW value. It is well known that an increased RDW value in a blood sample indicates the presence of anisocytosis; furthermore, it is also known that sheep have a physiological anisocytosis of RBCs [27]. RDW values in the blood samples of sheep in this study were within the physiological range [19], and the obtained results provide an insight into new findings relating to the distribution of the obtained RBC subpopulations depending on the higher or lower RDW value. The distribution of the obtained RBC subpopulations in the formed groups is difficult to compare and explain because the data from this research that relate to RBC morphometric parameters and RBC subpopulations were for the first time obtained and analysed in this manner. However, the degree of otherwise physiological anisocytosis changes in anaemic conditions. Those changes are likely to be accompanied by a different distribution of RBCs in subpopulations. Furthermore, the sheep in this study were clinically healthy, with all haematological parameters values within the reference interval for sheep [19]. Therefore, we believe that the results obtained in this research represent a proper basis for future evaluation of RBC morphometric parameters in sheep, especially in animals with haematological disturbances (anaemia, haemoparasites), and provide new insight into future research in this field.

## 5. Conclusions

This is, to the best of our knowledge, the first report on the morphometric parameters of sheep RBCs that includes differentiation into subpopulations and focuses on clinically healthy sheep outside of the reproductive season. Morphometric parameters of the size and shape of RBCs in Lika pramenka sheep appear to be the most representative to mark changes in the normal morphometry of RBCs. Furthermore, it can be concluded that ovine RBC subpopulations based on morphometric size and shape parameters can be identified using computer-assisted image analysis of RBC morphometry and multivariate statistical methods, including principal component and cluster analysis. However, data on RBC subpopulations are scarce or non-existent. Therefore, it would be of great interest in the future to compare RBC subpopulations among different sheep breeds or animals with different (patho) physiological status (anaemias, pregnancy, nutrition, metabolic diseases). RBC morphometry could prevent any potential confusion in studying anaemic syndromes, i.e., it could serve as a basis for diagnostic interpretation of anaemic syndromes in veterinary medicine, especially concerning normocytic, macrocytic, and microcytic anaemias in sheep. In conclusion, RBC morphometry may have important applications and become a part of standard procedures for diagnostic interpretation and treatment of haematological disorders in which the detection of ovine RBCs shape changes is crucial.

## Figures and Tables

**Table 1:** Morphometric size and shape parameters of red blood cells (RBCs) in Lika pramenka sheep (36 blood smears in toto were obtained from 36 sheep, with 4017 measured RBCs, approximately 110 from each smear).

Parameter		Mean	Median	Standard Deviation	Coefficient of Variation%	Minimaland Maximal Values	95% Confidence Interval	Variations between Sheep			
								Mean Value Interval	Median Value Interval	Standard Deviation Interval	Coefficient of Variation Interval%
**Rbcs’ Size Measures**	Area, µm^2^	21.19	20.96	3.45	11.95	11.17–48.97	21.09–21.30	16.86–25.62	16.67–25.57	2.16–4.41	4.69–19.48
	Outline, µm	17.32	17.26	1.45	2.12	12.72–27.49	17.28–17.37	15.46–18.99	15.40–18.92	0.94–1.94	0.88–3.78
	Convex	21.49	21.25	3.49	12.19	11.39–49.80	21.39–21.60	17.12–25.94	16.86–25.80	2.24–4.43	5.03–17.69
	Minimal Radius, µm	2.27	2.27	0.21	0.04	1.37–3.43	2.26–2.28	2.00–2.53	1.99–2.52	0.14–0.28	0.02–0.08
	Maximal Radius, µm	2.86	2.85	0.25	0.06	2.11–4.56	2.85–2.87	2.58–3.12	2.56–3.11	0.16–0.29	0.02–0.08
	Length, µm	5.51	5.49	0.47	0.22	3.90–8.92	5.49–5.52	4.93–6.03	4.93–5.98	0.31–0.58	0.09–0.34
	Breadth, µm	4.90	4.88	0.42	0.18	3.61–7.28	4.89–4.91	4.39–5.41	4.36–5.37	0.29–0.50	0.08–0.25
	Ellipticity	1.125	1.118	0.064	0.004	0.962–1.455	1.123–1.127	1.106–1.154	1.102–1.149	0.042–0.083	0.001–0.007
	Elongation	0.0584	0.0558	0.0278	0.0007	0.000–0.1855	0.0575–0.0592	0.0504–0.0702	0.0487–0.0696	0.0190–0.0355	0.0003–0.0012
	Solidity	0.985	0.988	0.008	0.000072	0.859–0.996	0.985–0.986	0.975–0.990	0.981–0.990	0.002–0.015	0.000006–0.000177
	Roundness	0.820	0.828	0.059	0.0035	0.459–0.955	0.818–0.822	0.764–0.855	0.766–0.863	0.037–0.079	0.0014–0.0062
	form Factor	0.882	0.889	0.035	0.001	0.405–0.932	0.881–0.883	0.852–0.896	0.857–0.900	0.013–0.115	0.000–0.013
	Contour Index	3.77	3.75	0.098	0.009	3.67–5.56	3.773–3.779	3.74–3.94	3.73–3.82	0.029–0.405	0.000–0.1647

Ellipticity = length/breadth; elongation = [(length − breadth)/(length + breadth)]; solidity = area/convex; roundness = (4 × area)/[ π × (major axis)2]; form factor = [4π × area/outline 2]; contour index = outline/√area.

**Table 2:** Comparison of the mean and standard error values of red blood cell (RBC) morphometric parameters between groups formed by categorization of the haematological parameter values in Lika pramenka sheep.

	Reference Values	Group	N Sheep	Group Mean ± STD	N Rbcs	RBC Mean ± Stderr						
						Area (µm^2^)	Outline (µm)	Convex	Minimal Radius (µm)	Maximal Radius (µm)	Length (µm)	Breadth (µm)
HGB g/L	90-150	HGB_1	7	84.67 ± 4.07	784	20.71 ± 0.12 ***	17.19 ± 0.05 *	21.01 ± 0.12 ***	2.236 ± 0.007 ***	2.839 ± 0.008 ***	5.45 ± 0.01 **	4.844 ± 0.015 ***
		HGB_2	29	99.91 ± 6.91	3233	21.31 ± 0.06	17.35 ± 0.02	21.65 ± 0.06	2.283 ± 0.003	2.870 ± 0.004	5.52 ± 0.01	4.919 ± 0.007
HCT	0.27-0.45	HCT_1	11	0.26 ± 0.01	1226	20.94 ± 0.09 *	17.21 ± 0.04 *	21.23 ± 0.10 *	2.260 ± 0.006 *	2.839 ± 0.007 ***	5.46 ± 0.01 ***	4.873 ± 0.012 *
		HCT_2	25	0.29 ± 0.01	2791	21.30 ± 0.06	17.37 ± 0.02	21.61 ± 0.06	2.280 ± 0.004	2.875 ± 0.004	5.53 ± 0.01	4.918 ± 0.008
MCV ƒL/cell	28.0-40.0	MCV_1	16	29.31 ± 1.15	1781	20.43 ± 0.08 ***	17.04 ± 0.03 ***	20.73 ± 0.08 ***	2.222 ± 0.005 ***	2.817 ± 0.005 ***	5.41 ± 0.01 ***	4.813 ± 0.009 ***
		MCV_2	20	31.40 ± 0.49	2236	21.80 ± 0.07	17.55 ± 0.03	22.10 ± 0.07	2.315 ± 0.004	2.900 ± 0.005	5.59 ± 0.01	4.978 ± 0.008
MCH pg/cell	8.0-12.0	MCH_1	14	9.82 ± 0.12	1563	20.40 ± 0.08 ***	17.03 ± 0.03 ***	20.71 ± 0.08 ***	2.222 ± 0.005 ***	2.815 ± 0.006 ***	5.41 ± 0.01 ***	4.815 ± 0.010 ***
		MCH_2	22	10.63 ± 0.29	2454	21.69 ± 0.06	17.51 ± 0.02	22.00 ± 0.07	2.307 ± 0.004	2.895 ± 0.004	5.58 ± 0.01	4.962 ± 0.008
MCHC g/L	310-340	MCHC_1	17	329.54 ± 7.60	1897	20.62 ± 0.07 ***	17.11 ± 0.03 ***	20.91 ± 0.08 ***	2.242 ± 0.005 ***	2.825 ± 0.005 ***	5.43 ± 0.01 ***	4.842 ± 0.009 ***
		MCHC_2	19	350.07 ± 9.50	2120	21.71 ± 0.07	17.51 ± 0.03	22.01 ± 0.07	2.303 ± 0.004	2.898 ± 0.005	5.58 ± 0.01	4.960 ± 0.009
RDW%	16-22	RDW_1	18	21.13 ± 0.43	2019	22.07 ± 0.07 ***	17.69 ± 0.03 ***	22.37 ± 0.07 ***	2.330 ± 0.004 ***	2.921 ± 0.005 ***	5.62 ± 0.01 ***	5.012 ± 0.009 ***
		RDW_2	18	22.75 ± 0.47	1998	20.30 ± 0.07	16.95 ± 0.01	20.60 ± 0.07	2.217 ± 0.004	2.805 ± 0.005	5.39 ± 0.01	4.796 ± 0.009
	**Reference Values**	**Group**	**N Sheep**	**Group Mean ± STD**	**N Rbcs**	**RBC Mean ± Stderr**						
						**Ellipticity**		**Elongation**	**Solidity**	**Roundness**	**form Factor**	**Contour Index**
HGB g/L	90–150	HGB_1	7	84.67 ± 4.07	784	1.128 ± 0.002		0.0592 ± 0.0009	0.9851 ± 0.0003 *	0.8149 ± 0.0021 *	0.8761 ± 0.0012 ***	3.795 ± 0.003 ***
		HGB_2	29	99.91 ± 6.91	3233	1.125 ± 0.001		0.0582 ± 0.0004	0.9860 ± 0.0001	0.8215 ± 0.0010	0.8840 ± 0.0006	3.771 ± 0.001
HCT	0.27–0.45	HCT_1	11	0.26 ± 0.01	1226	1.124 ± 0.001		0.0575 ± 0.0008	0.9863 ± 0.0002 *	0.8230 ± 0.0017	0.8829 ± 0.0010	3.777 ± 0.002
		HCT_2	25	0.29 ± 0.01	2791	1.126 ± 0.001		0.0587 ± 0.0005	0.9856 ± 0.0001	0.8194 ± 0.0011	0.8823 ± 0.0006	3.775 ± 0.001
MCV ƒL/cell	28.0–40.0	MCV_1	16	29.31 ± 1.15	1781	1.127 ± 0.001		0.0590 ± 0.0006	0.9850 ± 0.0002 ***	0.8155 ± 0.0014 ***	0.8786 ± 0.0008 ***	3.786 ± 0.002 ***
		MCV_2	20	31.40 ± 0.49	2236	1.124 ± 0.001		0.0579 ± 0.0005	0.9865 ± 0.0001	0.8239 ± 0.0012	0.8856 ± 0.0007	3.768 ± 0.002
MCH pg/cell	8.0–12.0	MCH_1	14	9.82 ± 0.12	1563	1.125 ± 0.001		0.0582 ± 0.0007	0.9852 ± 0.0002 **	0.8165 ± 0.0015 *	0.8789 ± 0.0008 ***	3.786 ± 0.002 ***
		MCH_2	22	10.63 ± 0.29	2454	1.126 ± 0.001		0.0585 ± 0.0005	0.9863 ± 0.0001 **	0.8226 ± 0.0012	0.8847 ± 0.0007	3.769 ± 0.001
MCHC g/L	310–340	MCHC_1	17	329.54 ± 7.60	1897	1.124 ± 0.001		0.0579 ± 0.0006	0.9858 ± 0.0001	0.8207 ± 0.0013	0.8813 ± 0.0008	3.780 ± 0.002*
		MCHC_2	19	350.07 ± 9.50	2120	1.126 ± 0.001		0.0588 ± 0.0006	0.9859 ± 0.0001	0.8197 ± 0.0013	0.8836 ± 0.0007	3.772 ± 0.002
RDW%	16–22	RDW_1	18	21.13 ± 0.43	2019	1.124 ± 0.001		0.0578 ± 0.0006	0.9864 ± 0.0001 ***	0.8221 ± 0.0013 *	0.8832 ± 0.0007	3.775 ± 0.002
		RDW_2	18	22.75 ± 0.47	1998	1.127 ± 0.001		0.0589 ± 0.0006	0.9852 ± 0.0001 *	0.8183 ± 0.0013	0.8817 ± 0.0007	3.776 ± 0.002

RBC—red blood cells; HGB—haemoglobin; HCT—haematocrit; MCV—mean corpuscular volume; MCH—mean corpuscular haemoglobin; MCHC—mean corpuscular haemoglobin concentration; RDW—red cell distribution width; ellipticity = length/breadth; elongation = [(length − breadth)/(length + breadth)]; solidity = area/convex; roundness = (4 × area)/[ π × (major axis)2]; form factor = [4π × area/outline2]; contour index = outline/√area; * *p* < 0.01; ** *p* < 0.001; *** *p* < 0.0001; reference values: [19].

**Table 3:** Eigenvalues of each red blood cell (RBC) morphometric variable for the three principal components (factors) in Lika pramenka sheep.

RBC Values	RBC Size	RBC Shape	
	Factor 1	Factor 2	Factor 3
Outline, µm	0.98 *		
Convex, µm^2^	0.98		
Area, µm^2^	0.97		
Length, µm	0.94		
Breadth, µm	0.92		
Roundness		0.86 *	
form Factor		0.80	
Contour Index		−0.75	
Elongation		−0.69	0.70 *
Ellipticity		−0.70	0.69
Solidity		0.68	
Characteristic ROOT (λ) and Explained Variance (%)	4.70 (42.8)	3.58 (32.5)	2.04 (18.5)
Cumulative Variance%	42.8	75.3	93.8

Ellipticity = length/breadth; elongation = [(length − breadth)/(length + breadth)]; solidity = area/convex; roundness = (4 × area)/[π × (major axis)2]; form factor = [4π × area/outline 2]; contour index = outline/√area; * the most important RBC value for each factor. Values higher than 0.60 are shown for each factor.

**Table 4:** Red blood cell (RBC) subpopulations based on the most important RBC value for each factor (mean and standard error) in Lika pramenka sheep.

RBC Subpopulation (Cluster)		RBC Size	RBC Shape	
	N (%)	Outline, µm	Roundness	Elongation
ES 1	778 (19.5)	15.38 ± 0.59	0.814 ± 0.059	0.059 ± 0.029
ES 2	915 (22.9)	19.11 ± 0.73	0.823 ± 0.058	0.057 ± 0.025
ES 3	2292 (57.6)	17.21 ± 0.73	0.820 ± 0.060	0.058 ± 0.027

ES 1—the smallest and most elongated RBCs; ES 2—the biggest and most rounded RBCs; ES 3—average size and shape RBCs; roundness = (4 × area)/[π × (major axis)^2^]; elongation = [(length − breadth)/(length + breadth)]; bold values—the highest or lowest value.

**Table 5:** Proportion of RBC subpopulations (ES 1—the smallest and most elongated RBCs; ES 2—the biggest and most rounded RBCs; ES 3—average size and shape RBCs) in Lika pramenka sheep grouped according to the values of the haematological parameters.

	Reference Values [19]	Group	Group Description	RBC Subpopulation %			Chi-Square Value	*p*-Value
				ES 1	ES 2	ES 3		
HGB g/L	90–150	HGB_1	≤90	26.1	20.8	53.1	26.477	<0.0001
		HGB_2	>90	17.9	23.5	58.6		
HCT	0.27–0.45	HCT_1	≤0.27	25.8	21.2	53.0	43.855	<0.0001
		HCT_2	>0.27	16.8	23.7	59.5		
MCV ƒL/cell	28.0–40.0	MCV_1	≤30	29.1	17.4	53.5	200.664	<0.0001
		MCV_2	>30	11.9	27.4	60.7		
MCH pg/cell	8.0–12.0	MCH_1	≤10	28.3	17.0	54.7	141.439	<0.0001
		MCH_2	>10	14.0	26.7	59.3		
MCHC g/L	310–340	MCHC_1	<340	21.6	16.9	61.5	76.274	<0.0001
		MCHC_2	≥340	17.6	28.5	53.9		
RDW%	16–22	RDW_1	≤22	10.0	29.0	61.0	260.308	<0.0001
		RDW_2	>22	29.2	16.8	54.0		

HGB—haemoglobin; HCT—haematocrit; MCV—mean corpuscular volume; MCH—mean corpuscular haemoglobin; MCHC—mean corpuscular haemoglobin concentration; RDW—red cell distribution width; reference values [19]; group description: groups were formed by categorizing continuous variables of analysed values of haematological parameters (HGB, HCT, MCV, MCH, MCHC, and RDW) by categorising them into two groups—those with lower values or values below the physiological range (Groups 1), and those with higher values or values above the physiological range (Groups 2), bearing in mind that the groups have approximately the same number of samples each.
